# Non-Invasive Transcriptomic Profiling of Inflammatory Skin Diseases Using Four Tape Strips

**DOI:** 10.3390/ijms27198544

**Published:** 2026-09-24

**Authors:** Anna Schultz Vinge, Sule Altintas, Niels Ødum, Jeanne Duus Johansen, Lone Skov, Marianne Bengtson Løvendorf

**Affiliations:** 1 Department of Dermatology and Allergy, Copenhagen University Hospital, Herlev and Gentofte, 2900 Hellerup, Denmark; anna.schultz.vinge@regionh.dk (A.S.V.);; 2 Department of Clinical Medicine, Faculty of Health and Medical Sciences, University of Copenhagen, 2200 Copenhagen N, Denmark; 3 The Leo Foundation Skin Immunology Research Center, Department of Immunology and Microbiology, Faculty of Health and Medical Sciences, University of Copenhagen, 2200 Copenhagen N, Denmark; 4 The National Allergy Research Centre, Department of Dermatology and Allergy, Copenhagen University Hospital, Herlev and Gentofte, 2900 Hellerup, Denmark

**Keywords:** atopic dermatitis, contact dermatitis, psoriasis, RNA, transcriptomics, skin tape

## Abstract

Tape strip-based transcriptomics shows promise for precision medicine in inflammatory skin diseases, but methodological variation limits reproducibility and clinical use. This study investigates whether four or eight tape strips are sufficient for transcriptomic profiling across three inflammatory skin diseases. As part of the Danish cohort study BIOSKIN, patients with atopic dermatitis (AD), contact dermatitis (CD), psoriasis, and healthy controls (n = 8 per group) were enrolled. Two sets of eight consecutive tape strips were obtained from each participant from lesional skin (patients) or healthy skin (controls) and pooled for RNA sequencing as strips 1–8, 1–4, or 5–8. Despite low RNA yield, sequencing was successful across all disease groups. Gene detectability was consistent across tape strip strategies, with 78.1%, 73.1%, and 67.7% overlap across strips 1–4, 5–8, and 1–8 for psoriasis, AD, and CD, respectively. Principal component analysis distinguished patients with psoriasis and AD from healthy controls, and enrichment analysis captured biologically relevant pathways across all three disease groups. In psoriasis, tape strip transcriptomics captured a core psoriatic transcriptome consistent with paired biopsy data. Four tape strips were sufficient for RNA sequencing and detection of disease-relevant inflammatory responses, supporting their use in longitudinal cohort studies and split-sampling designs. Performance was most robust for psoriasis and AD; optimization may benefit CD given the challenges of palmar sampling.

## 1. Introduction

Psoriasis, atopic dermatitis (AD), and contact dermatitis (CD) are chronic inflammatory skin diseases. Psoriasis is an autoimmune condition driven by T-helper 17 (Th17) cell responses [1], while AD is associated with a Th2-skewed immune profile and impaired skin barrier function [2]. Patients with CD represent a heterogeneous group covering different etiologic subtypes such as allergic or irritant contact dermatitis (ACD, ICD) with or without comorbid AD [3]. Thus, overlapping profiles in immune dysregulation are observed between AD and CD—even across different etiologic subtypes of CD [4]. Shared inflammatory mediators have also been described in AD and psoriasis despite different mechanisms of pathogenesis [5].

Transcriptomic profiling has revolutionized dermatology research by enabling in-depth molecular characterization of inflammatory skin diseases [4,6,7]. Traditionally applied to skin biopsies, this approach has provided key insights into disease-specific immune pathways and potential therapeutic targets. Yet, skin biopsies are limited by the invasive nature of the procedure, patient discomfort, and the risk of scarring. These constraints are particularly relevant in contexts where repeated sampling is required, such as longitudinal cohort studies and clinical trials monitoring treatment response, pediatric patients, or where the affected anatomical site renders biopsy impractical, as is the case for skin diseases on the hand and foot, including CD [8,9,10].

Tape stripping offers a rapid, painless, and patient-friendly alternative for sampling the epidermis from the stratum corneum to the stratum granulosum [8], enabling access to both proteomic and transcriptomic information from the same sample [11,12,13,14,15]. These properties make tape stripping particularly ideal for large-scale biobanking and longitudinal study designs requiring repeated, non-invasive sampling. However, the low RNA yield and susceptibility to degradation inherent to tape strip samples remain a persistent challenge, and standardized protocols are currently lacking. The number of tape strips collected varies widely across studies, with some protocols using up to 20 or 30 strips [13,16], making cross-study comparisons difficult and limiting practical implementation.

Within the BIOSKIN cohort, a longitudinal biobank program for inflammatory skin diseases, eight consecutive tape strips are collected from each patient per visit [17]. We have previously shown that four of these tape strips are sufficient for targeted proteomic analysis using the Olink platform across AD, CD, and psoriasis, as well as in palmoplantar inflammatory skin diseases [18,19]. Whether the remaining four tape strips yield sufficient and interpretable transcriptomic signal is an open question with direct practical implications for split-sampling strategies in cohort settings.

This study aims to determine whether four tape strips, or the full eight, are sufficient for transcriptomic investigation of psoriasis, AD, and CD. For a subset of patients with psoriasis, we compared tape strip transcriptomics with paired skin biopsy data to evaluate if tape strips capture concordant biology.

By establishing a resource-efficient protocol for tape strip-based transcriptomics, we aim to facilitate the wider adoption of this method in research and clinical settings, ultimately improving our ability to study inflammatory skin diseases in a less invasive and more scalable and patient-friendly manner.

## 2. Results

The study included a total of 32 participants, comprising 8 patients with AD, CD, and psoriasis, respectively, and 8 healthy controls. Baseline characteristics for all participants and local severity scores for patient groups are presented in Table 1.

### 2.1. Four Tape Strips Supported RNA Sequencing Across Disease Groups Despite Low RNA Yield

Overall quality of the tape strips for each group is presented in Figure 1b–d (Table S1). The total RNA concentration and quality were low across all groups of tape strips (Figure 1b). The highest RNA concentration was found in tape strips from patients with psoriasis (median (inter-quartile range (IQR)) = 2.1 ng/µL (0.9–3.7)), followed by patients with AD (median (IQR) = 0.4 ng/µL (0.2–1.3)). Tape strips from patients with CD and healthy controls had the lowest RNA concentration, with overall medians (IQR) of 0.08 ng/µL (0.04–0.4) for CD and 0.03 ng/µL (0.02–0.05) for controls. Most samples showed a mapping rate below 70% (Figure 1c), a threshold often used to define high-quality RNA sequencing data. This was expected given the low RNA yield and partial degradation inherent to tape stripping (Table S1). Only samples from patients with psoriasis achieved a median mapping rate above 70%, likely reflecting their higher RNA concentrations. Despite overall low RNA quality, a substantial number of genes were detected across all groups, demonstrating that tape strips are suitable for high-throughput RNA sequencing (Figure 1d). Notably, comparable results were obtained whether RNA was extracted from four or eight pooled tape strips (Figure 1b–d), supporting the feasibility of this minimally invasive sampling method for clinical transcriptomic studies.

### 2.2. Tape Strip-Based Transcriptomics Effectively Distinguish Patients with Psoriasis and Atopic Dermatitis from Healthy Controls

To assess the overall structure and biological drivers of variation in our dataset, we performed a principal component analysis (PCA) and evaluated the influence of different variables on gene expression. The PCA revealed partially overlapping but distinguishable clusters for psoriasis and AD samples, while CD samples clustered closely with healthy controls (Figure 2a).

To identify the main contributors to gene expression variability, we evaluated the impact of several clinical and technical factors. The disease group accounted for the largest proportion of variance, underscoring the ability of tape strip-based transcriptomics to capture meaningful biological differences between skin diseases (Figure 2b). This was followed by the anatomical location of sampling (sample site)—a known source of variation in skin transcriptomics [4] and RNA concentration [20]. In contrast, the number of tape strips used (1–4, 5–8, or 1–8) had minimal influence on variance, as did participant age and sex.

### 2.3. Identifying a Shared Core Set of Transcripts Across Different Tape Stripping Strategies and Disease Groups

We next sought to evaluate whether different tape stripping strategies captured a shared core set of transcripts. For each disease group, we compared genes detected using tape strips 1–4, 5–8 and 1–8; the overlap was visualized in Venn diagrams (Figure 2c, Tables S2–S4). Overlapping detected genes across these strategies accounted for 73.1% of all detected genes in AD, 67.7% in CD, and 78.1% in psoriasis, indicating a high degree of consistency in gene detection regardless of which tape strips were used. As presented in Figure 2d, the UpSet plot revealed a striking number of detected genes shared across different tape stripping strategies as well as the three disease groups, including pro-inflammatory mediators such as cytokines, interleukins, and chemokines. The second- and third-largest intersections were shared across different tape stripping strategies within the psoriasis group and between psoriasis and AD, respectively. Together, these findings support the robustness of the method and reinforce that using only four tape strips is sufficient for detecting a broad range of relevant genes.

### 2.4. Confirmation of Disease-Specific Molecular Signatures in Tape Strips

To ensure molecular signatures in each inflammatory skin disease were captured in tape strip-based transcriptomics, differential expression analysis was performed comparing each disease group with healthy controls. Expression levels of selected disease-relevant genes based on existing literature [4,10,13,21,22] are presented in Figure 3.

In patients with AD, we found upregulation of well-known genes such as CCL17 involved in Th2-inflammation, and the innate chemokine CXCL8 along with the serine protease inhibitor SERPINB4 and matrix metalloproteinase (MMP) 12, a marker of general inflammation. IL-13, a key interleukin in the pathogenesis of AD, was not differentially expressed compared to healthy controls.

In patients with psoriasis, we found an upregulation of molecular features such as IL-17A, IL-36A, S100 proteins (S100A8 and A9), defensins (DEFB4A), and regulation of the inflammatory nuclear factor kappa B (NF-κB) signaling pathway (NFKBIZ). In the group with CD, we found upregulation of different proinflammatory mediators, such as IL-1β, members of the NF-κB signaling pathway (NFKBIZ and NFKB1), as well as MMP1 and 12 (Figure 3), both involved in TNF-driven inflammation [23].

### 2.5. Enriched Immunologic Pathways Are Well-Captured in Tape Strips

To assess whether relevant immunologic pathways were captured in tape strips, we performed gene set variation analysis on gene sets from Gene Ontology Biological Processes. A wide range of immunologic pathways were significantly enriched in each disease group compared to healthy controls, with tape strips 1–4, 5–8, and 1–8 combined within each group, comprising acute and chronic inflammatory response, innate and adaptive immune response, cytokine production and signaling, T helper cell differentiation, along with chemotaxis and migration of various immune cells. GSVA enrichment scores for selected pathways are depicted in Figure 4. For enrichment scores of all pathways in each disease group vs. controls, please refer to Tables S5–S7.

### 2.6. Tape Strips 5–8 Capture More Enriched Pathways and Larger Effect Sizes in Enrichment Scores than Tape Strips 1–4

After establishing that four tape strips were sufficient for whole transcriptomics, we sought to establish whether tape strips 1–4 or 5–8 were most suitable. When investigating DEGs between the two sample groups within each disease group, no significant differences in expression profiles were found.

Next, enriched pathways in disease groups vs. controls found in tape strips 1–4 were compared to those enriched in tape strips 5–8. In patients with psoriasis, the majority of significantly enriched pathways at 5% FDR (separate Benjamini–Hochberg adjustment) were captured in tape strips 5–8 (182 and 259 enriched pathways in tape strips 1–4 and 5–8, respectively). However, after correcting for multiple testing globally across contrasts, 32 and 40 pathways were significantly enriched in tape strips 1–4 and 5–8, respectively (Table S8). Overlapping biological signals detected in both sample types included pathways related to angiogenesis, MAPK/NF-κB-signaling and lipid metabolism. Although large effect sizes (ΔGSVA enrichment scores) were captured in both sample types across numerous relevant pathways related to Th17 differentiation, keratinocyte differentiation, toll-like receptor signaling and broad inflammatory pathways when comparing to healthy controls, none of these were significant after multiple testing correction.

Considering patients with AD, thousands of significantly enriched pathways were identified in both sample groups based on unadjusted *p*-values. With separate Benjamini–Hochberg adjustment, we detected 1760 pathways significantly enriched at 5% FDR, including Th2 differentiation in tape strips 5–8, whereas no pathways were enriched in tape strips 1–4 (Table S9). When taking global adjustment for multiple testing across contrasts into account, tape strips 5–8 outperformed tape strips 1–4 with 110 significantly enriched pathways vs. 12, respectively. Biological signals captured in tape strips 5–8 included leukocyte aggregation, cytokines, pattern recognition signals, and pathways within keratinocyte and skin barrier function. Tape strips 1–4 did, however, capture significant enrichment in regulation of type 2 immune response. In patients with CD, only one enriched pathway in tape strips 1–4 (CD40 signaling) and 5–8 (phosphatidylethanolamine metabolic process) reached global significance after multiple testing correction (Table S10).

Larger effect sizes (ΔGSVA enrichment scores when comparing disease group with healthy controls) were captured in tape strip 5–8 compared to tape strips 1–4 in AD (Figure S1), yet, when directly comparing differences in ΔGSVA enrichment scores between tape strips 1–4 and 5–8 ((disease group_1–4_-HC_1–4_)-(disease group_5–8_-HC_5–8_)), no statistically significant differences were found in either of the disease groups.

### 2.7. Tape Strip Transcriptomics Captures Core Disease-Relevant Gene Expression in Psoriasis Compared to Skin Biopsies

To contextualize the biological signal captured by tape strips, we compared transcriptomic profiles from tape strips (1–4, 5–8, and 1–8) with paired skin biopsies obtained from a subset of patients with psoriasis (n = 4). As expected, biopsies yielded higher RNA concentrations (median = 215 ng/µL (IQR: 201–223) vs. median = 2.1 ng/µL (IQR: 0.9–3.7), respectively) and superior RNA integrity numbers (RINs) (median = 7.5 (IQR: 7.0–8.1) vs. median = 3.1 (IQR: 2.0–4.3)) compared to tape strips (Figure S2). Nevertheless, the mapping rate of reads was acceptable across all sample types, with a median of 86% (IQR: 86–91%) for biopsies and 73% (IQR: 56–87%) for tape strips.

Notably, the biotype distribution differed: Biopsy-derived reads were enriched for protein-coding transcripts, while tape strips exhibited mixed RNA species, potentially due to degraded RNA fragments being less well annotated (Figure 5a, Table S11). Although many dermis-derived collagens such as COL3A1 were detectable in all sample types, expression was higher in biopsy specimens compared to tape strips, as expected [24,25], whereas no difference was observed with regard to filaggrin, which is primarily located in the stratum corneum (Figure 5b). Notably, a key inflammatory cytokine in psoriasis, IL-17F, was increased in all sample types compared to healthy controls, along with KLK6 and GLUL, both recently found to be upregulated in the outer epidermis of patients with psoriasis [26].

The overlap in detected genes across biopsies and tape strips was substantial, with 52.8% of genes detected across both sample types (Figure S3, Table S12). When comparing psoriasis to healthy controls, 222 DEGs were consistently identified across biopsies and all tape strip combinations (Tables S13 and S14), representing a shared core psoriatic transcriptome that included well-established psoriasis-associated genes such as IL-36A/G, S100A9, CCL20, STAT3, and SERPINB4 [13] (Figure 5c, Table S13). IL-17A was differentially expressed in psoriatic skin compared to healthy controls only in tape strips 5–8 and biopsies (Table S13). As expected, biopsies yielded additional DEGs not detected in tape strips, reflecting their capacity to capture a broader tissue compartment (e.g., IFN-γ, CXCL10, IL-20 and IL-12B). Notably, tape strips also identified a set of DEGs unique to this sampling method (3582 and 3504 upregulated DEGs in tape 5–8 and 1–4, respectively; Figure 5c, Table S13). This included NFKB1, MAPK3/8/9/10/13, PIK3CB, PIK3C2B, and TBK1 related to NF-κB, MAPK, PI3K and mTOR signaling, as well as IL-36B (Table S13). While some of these may represent genuine stratum corneum-specific biology, this finding should be interpreted with caution given the fragmented nature of tape strip RNA, which may introduce uncertainty in transcript annotation.

## 3. Discussion

In this feasibility study, we found that four tape strips were sufficient to support RNA sequencing for patients with AD, CD, and psoriasis with the most robust performance in psoriasis and AD. In CD, results should be interpreted with caution due to lower RNA yield and fewer detected genes, likely reflecting the challenges of sampling palmar skin. Nevertheless, thousands of genes and enriched pathways were detected, which is encouraging given that CD predominantly manifests at sites where non-invasive sampling is particularly warranted. We found a convincing number of detected genes to be shared across all three inflammatory skin diseases. Similar to our results, previous research has demonstrated mutual inflammatory pathways and molecular features despite different disease mechanisms and pathogenesis; Tsoi et al. found 81% of dysregulated genes in lesional AD to be in common with those in psoriatic skin [5]. Correspondingly, Quaade et al. showed that a high number of DEGs in hand eczema patients were shared across AD, ICD and ACD [4]. Thus, detecting a shared core transcriptome was consistent with our expectations.

Based on comparison of GSVA enrichment scores with the control group, we found three different Th-differentiation pathways consistently upregulated across all disease groups: Th1, Th2, and Th17. Although the prevailing view considers AD as a Th2-skewed disease and psoriasis as Th17-skewed, enrichment of Th1, Th2 and Th17-pathways in both patients with AD and psoriasis has previously been demonstrated by Tsoi et al. [5]. Considering two recent studies investigating the transcriptome of chronic hand eczema, upregulation of Th1 and Th2 was expected in our patients with CD [4,10]. In contrast, Th17 has been suggested to play only a limited role in hand eczema [4] and was even found to be downregulated compared to controls in one study—this was, however, only significant in non-lesional skin [10]. Hence, we were surprised to find Th17 to be more significantly upregulated than Th2 in patients with CD.

When choosing which layers to use for transcriptomic analysis, one might hypothesize that the outcome of using either tape 1–4 or 5–8 would be indistinguishable based on the results by Clausen et al., showing that stable cytokine concentrations were demonstrated across different tape strips, supporting a view of stratum corneum as having a consistent molecular milieu [27]. In contrast to this, we showed that choosing either tape 1–4 or 5–8 impacted the capability of detecting significantly enriched inflammatory pathways. In this manner, tape 5–8 outperformed tape 1–4 when investigating patients with psoriasis and AD. However, when directly testing tapes 1–4 vs. 5–8, no difference-in-differences was statistically significant in either patient group compared with healthy controls, consistent with the findings of Clausen et al. [27]. We considered this an appropriate opportunity to examine the correlation between concentrations from the targeted inflammatory proteomic panel [18] and gene expression in patients that appeared in both studies—boxplots of overall correlation in each group are shown in Figure S4. The two methods correlated to a moderate degree regarding patients with psoriasis and AD. In the case of CD and healthy controls, we saw a poor correlation between the two methods, yet it must be considered that several protein concentrations were either in proximity to or below the detection limit in these groups, and thus the data must be cautiously interpreted. Furthermore, protein concentration and gene expression do not necessarily reflect each other [28].

A key strength of this study is the investigation of the same streamlined protocol for tape strip-based transcriptomics in three different inflammatory skin diseases. To the best of our knowledge, these three patient groups have not been investigated side-by-side in a similar set-up. Furthermore, we incorporated a direct comparison of tape strips and biopsies in a patient subgroup to further elaborate on what is captured by the two methods. The findings of this study should be considered in light of its design including a small sample size and variation in anatomical sample sites. CD represented a clinically heterogeneous group, and samples were predominantly obtained from palmar skin, which is the primary site of disease manifestation and a location where non-invasive sampling is particularly advantageous. As gene expression varies across anatomical sites [4], direct comparison with samples from other body regions, as used for psoriasis, AD, and healthy controls, introduces a degree of site-related variability. Future studies including site-matched healthy controls for CD would strengthen the interpretation of findings in this group. Furthermore, only eight patients were enrolled in each group, and lack of significance in CD patients could be due to a small sample size.

Of note, we identified a compelling number of DEGs in tape strips compared to biopsies—this tendency was accentuated when using four tape strips instead of eight. These results were in line with the studies by Tsoi et al. and Fritz et al. comparing tape strips and biopsies in patients with psoriasis and AD, respectively; they both found more DEGs in tape strips than biopsies [25,29]. Consistent with our results, this was found despite lower RNA yield and lower mapping rate in tape strips compared to biopsies [25]. Specifically, Tsoi et al. [29] reported ~28% of reads assigned to gene-level features in tape strips versus ~93% in biopsies [25], comparable to the mapping rates we observed. This lower RNA yield and degree of degradation reflect a persistent challenge with tape-strip sampling. Tam et al. have hypothesized that RNA is nonetheless obtainable in tape strips because it originates from epidermal and dermal turnover, being transported to the epidermis with dead or dying cells [30].

In conclusion, we showed that four tape strips were sufficient for whole transcriptomic analysis of lesional skin from patients with AD, CD, and psoriasis. The applied method appeared particularly well-suited to patients with AD and psoriasis, whereas protocol optimization may further improve its performance in CD. The lack of significance in the CD group could reflect the absence of site-matched control samples rather than a true absence of biological effect. Despite this, enrichment analysis captured biologically relevant pathways across all three disease groups, supporting the overall use of tape-strip sampling for transcriptomic profiling of inflammatory skin disease.

## 4. Materials and Methods

### 4.1. Study Population

This study was conducted as part of the Copenhagen Translational Skin Immunology Biobank and Research Program (BIOSKIN) [17]. Patients ≥ 18 years with psoriasis (n = 8), AD (n = 8), CD (n = 8), and healthy controls (n = 8) were recruited from November 2023 to January 2024 from the Department of Dermatology and Allergy, Gentofte Hospital, Denmark. Some participants also appear in our study evaluating a targeted proteomic approach (Olink) for tape strips [18].

### 4.2. Sample Collection

A total of two sets of eight consecutive tape strips were obtained from each participant from two adjacent areas of the same lesion (Figure 1a). At the first site, strips 1–8 were collected as a full-depth sample. At the second site, tape strips 1–4 (superficial stratum corneum layers) and tape strips 5–8 (deeper layers) were collected sequentially and kept separate. Severity of all lesions was evaluated using a local severity score (Table 1). Tape strips were pooled before transcriptomic analysis as either the full depth (strips 1–8), superficial layers (strips 1–4), or deeper layers (strips 5–8). For comparison, skin punch biopsies (2 mm) were collected under local anesthesia (lidocaine + adrenaline, 20 mg+5 µg/mL) from psoriatic lesional skin (n = 4) and healthy skin (n = 4) from the same individuals as the tape strip donors; the sampling site was concordant in 7 of 8 pairs. Detailed descriptions of sample collection, RNA isolation, library preparation, and RNA sequencing can be found in the Supplementary Materials.

### 4.3. Processing of Sequencing Data, Bioinformatic and Statistical Analysis

Processing of sequencing data and bioinformatic and statistical analysis are described in detail in the Supplementary Material. In short, RNA sequence reads were mapped to the human reference genome GRCh38 using Qiagen’s RNA-seq Analysis Portal 4.0 [31]. Gene name and biotype annotations were retrieved from Ensembl using biomaRt version 2.64.0 [32]. All statistical analyses were carried out in R versions 4.3.0 and 4.4.1. Venn diagrams were generated using systemPipeR version 2.16.4 [33] and ggvenn version 0.1.19 [34] and recreated by hand. Principal component analysis (PCA) was performed using DESeq2 version 1.42.1 [35], and variance partitioning using the variancePartition library [36]. Differentially expressed genes (DEGs) were identified using DESeq2 with an absolute fold change (FC) threshold of 1.5 and a 5% false discovery rate (FDR). An UpSet plot was generated with the ComplexUpset package version 1.3.3 [37,38]. Gene set variation analysis [39] was performed on GO Biological Processes gene sets [40]. We identified differentially expressed pathways at 5% FDR using limma [41]. When investigating the separate contrasts (disease group_1–4_-HC_1–4_) and (disease group_5–8_-HC_5–8_) along with ((disease group_1–4_-HC_1–4_)-(disease group_5–8_-HC_5–8_)), a global correction across contrasts was applied to ensure appropriate adjustment for multiple testing.

Some of the study participants were also included in a separate study [18], where tape strips were analyzed with the Olink Target Inflammation panel. Samples were matched by participant, condition, and tape-strip group. For genes measured by both platforms (87 genes), we calculated the Pearson correlation between vst-normalized RNA counts and Olink NPX values within each sample, across genes.

## Figures and Tables

**Figure 1:**
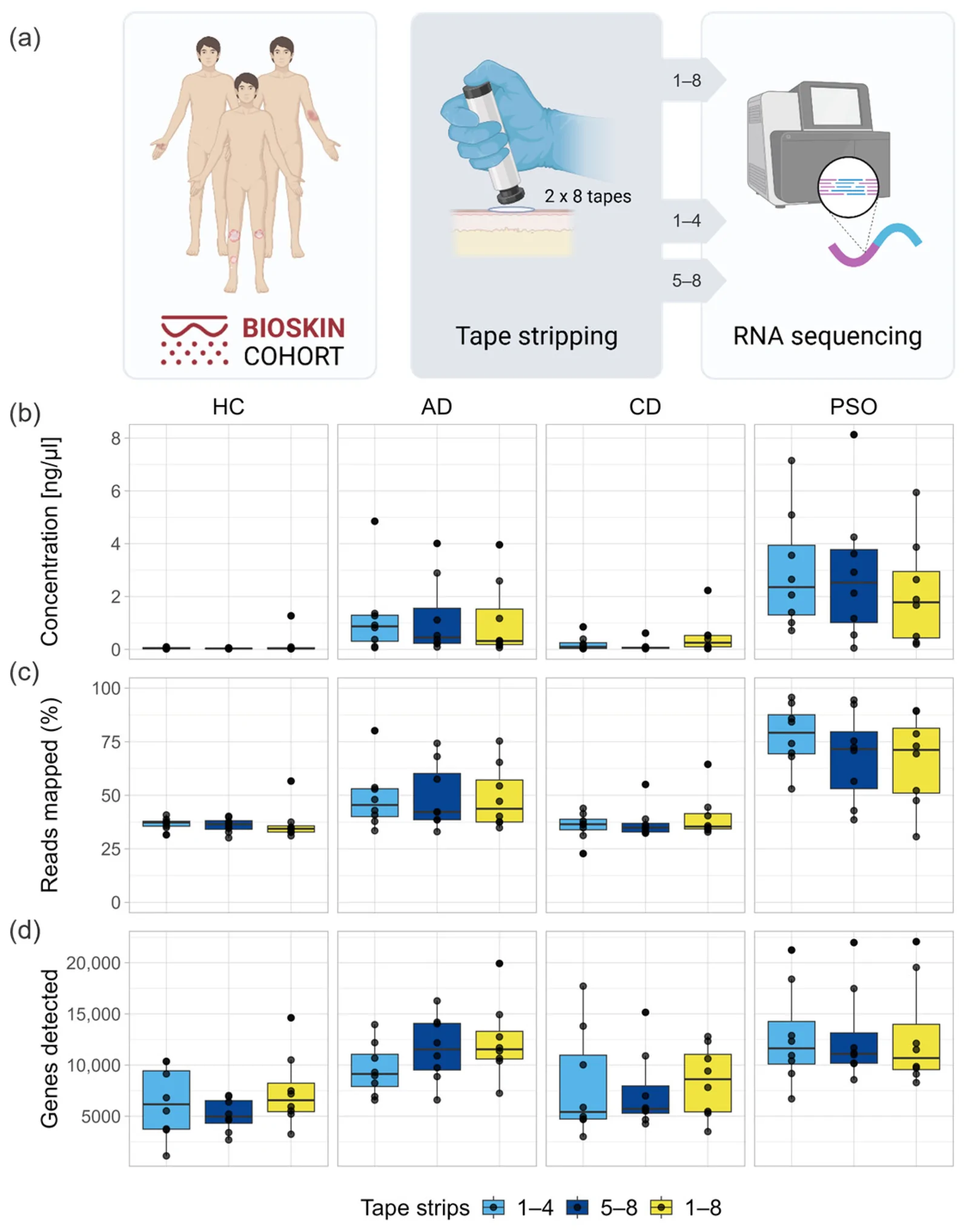
(**a**) Overview of study design, created with BioRender.com (**b**) Boxplots depicting concentrations of RNA isolated from tape strips. (**c**) Boxplots of the proportion of reads aligned to the reference genome (**d**) Boxplots of the number of genes detected (a gene was counted as detected if it had an average gene count of at least 10 across the samples). Abbreviations: AD, atopic dermatitis; CD, contact dermatitis; HC, healthy controls; PSO, psoriasis.

**Figure 2:**
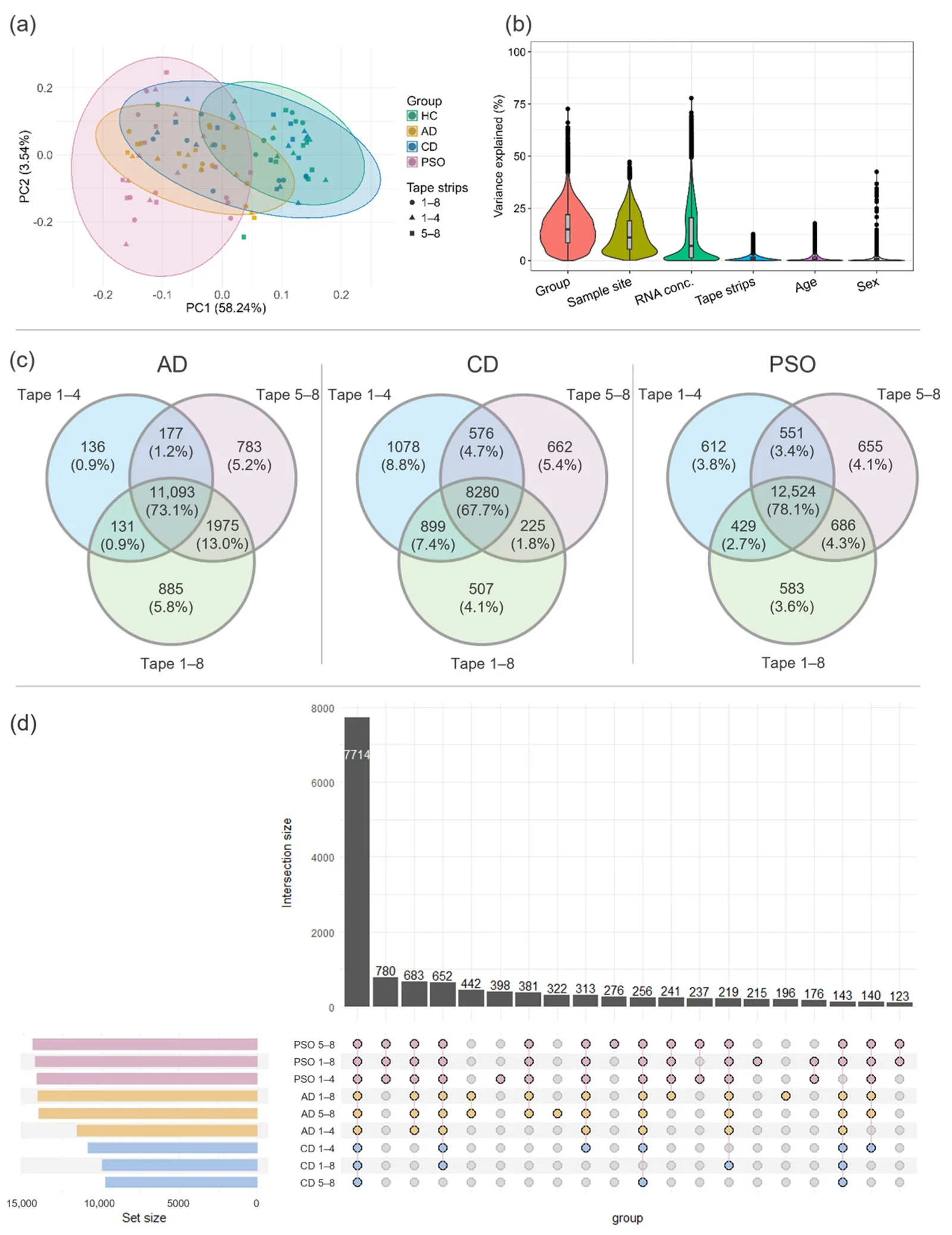
(**a**) Principal component analysis (PCA) of normalized gene expression profiles for the different patient groups and healthy controls with their 95% confidence intervals. (**b**) Gene-level variance explained by patient condition (AD, CD, psoriasis or HC), sex, and age; the sample site for the tape strips; number of tape strips used; and the sample RNA concentration. (**c**) Venn diagrams depicting the number of genes detected (mean n ≥ 10) in different tape strips (1–4, 5–8, and 1–8) from patients with AD, CD, and psoriasis. (**d**) UpSet plot showing top 20 intersections of detected genes (mean n ≥ 10) in different tape strips (1–4, 5–8, and 1–8) from patients with AD, CD, and psoriasis. Horizontal bars present the total number of detected genes in a tape strip sample group (1–4, 5–8, 1–8) for each disease group (AD, CD, psoriasis). Vertical bars present intersection scores indicating the number of detected genes shared among groups with filled dots in the corresponding matrix below the bar plot. Abbreviations: AD, atopic dermatitis; CD, contact dermatitis; HC, healthy controls; PC, principal component; PSO, psoriasis.

**Figure 3:**
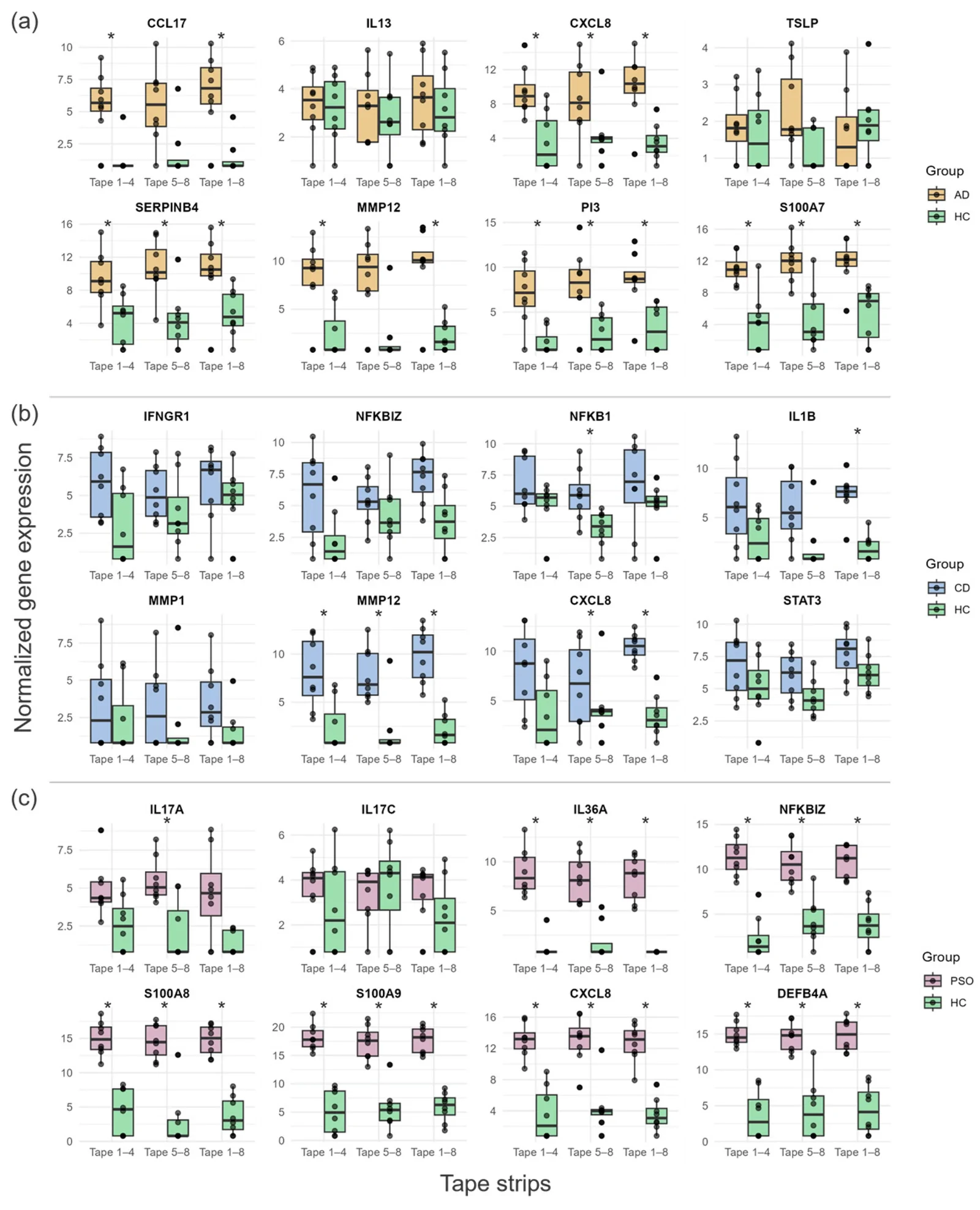
Normalized gene expression of selected disease-relevant genes in tape strips from patients with AD (**a**), CD (**b**), and psoriasis (**c**) compared to healthy controls. Dots show individual samples, horizontal lines represent medians with lower and upper hinges representing the 25th and 75th percentiles, and whiskers reach to the most extreme data point (maximum 1.5 times the IQR). Gene expression levels in each tape strip sample group (1–4, 5–8 and 1–8) were compared to the corresponding sample group for healthy controls and significant differences in |FC| > 1.5 at 5% FDR are marked with *. Abbreviations: AD, atopic dermatitis; CD, contact dermatitis; HC, healthy controls; PSO, psoriasis.

**Figure 4:**
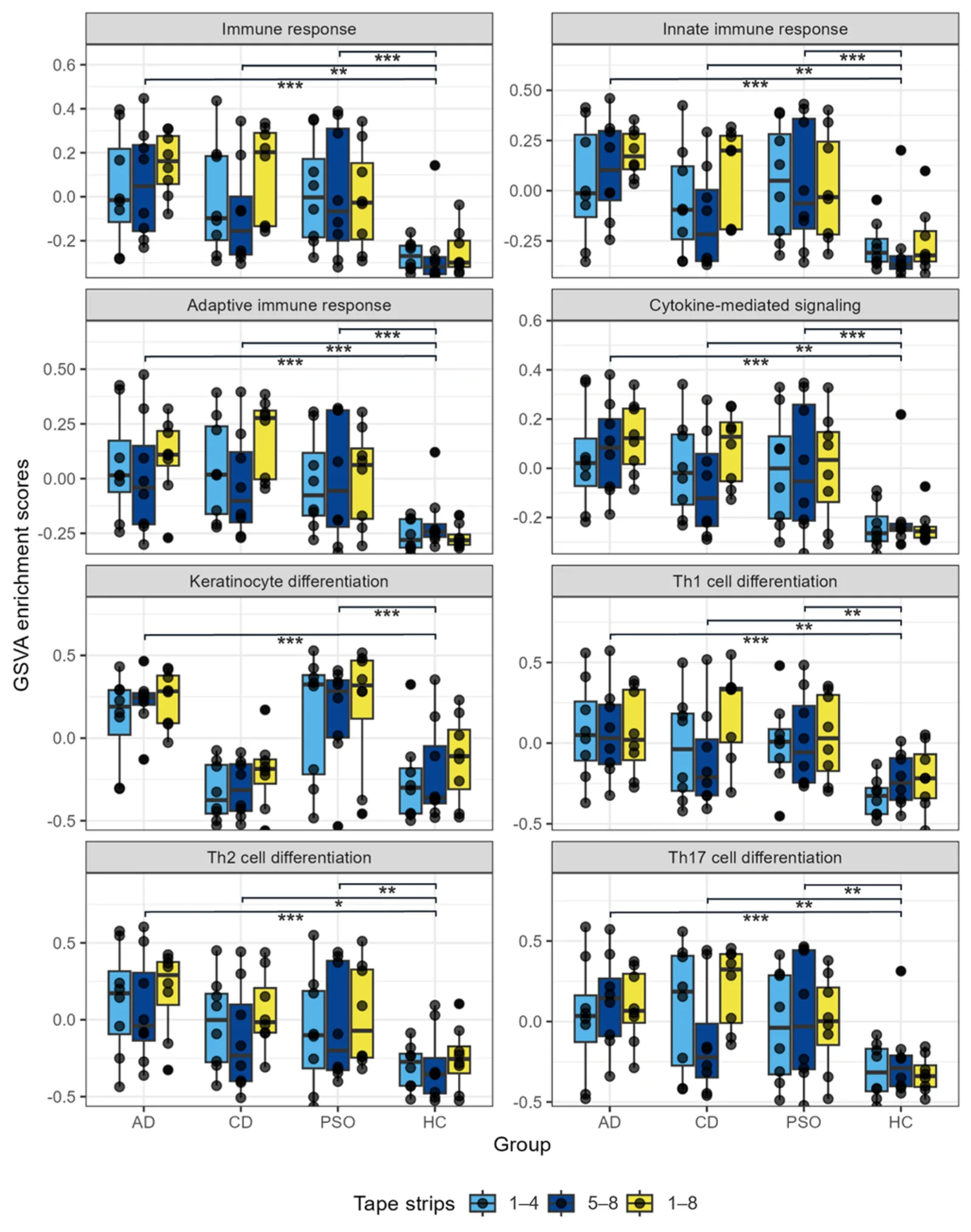
GSVA enrichment scores in patients with AD, CD, psoriasis and healthy controls in selected pathways from Gene Ontology Biological Processes. Dots show enrichment scores in individual samples; horizontal lines represent medians with lower and upper hinges representing the 25th and 75th percentiles and whiskers reaching the most extreme data point (maximum 1.5 times the IQR). For each gene set, enrichment scores in tape strips from each disease group (AD, CD, PSO; tape strips 1–4, 5–8, and 1–8 combined) were compared to healthy controls (HC) (tape strips 1–4, 5–8, and 1–8 combined) using moderated t-statistics. *, FDR < 0.05; **, FDR < 0.01; ***, FDR < 0.001. Gene set names (number of genes): activation of immune response (621), activation of innate immune response (341), adaptive immune response (765), cytokine-mediated signaling pathway (552), keratinocyte differentiation (179), Th1 cell differentiation (24), Th2 cell differentiation (18), Th17 cell differentiation (41). Abbreviations: AD, atopic dermatitis; CD, contact dermatitis; HC, healthy controls; PSO, psoriasis; Th, T helper cell.

**Figure 5:**
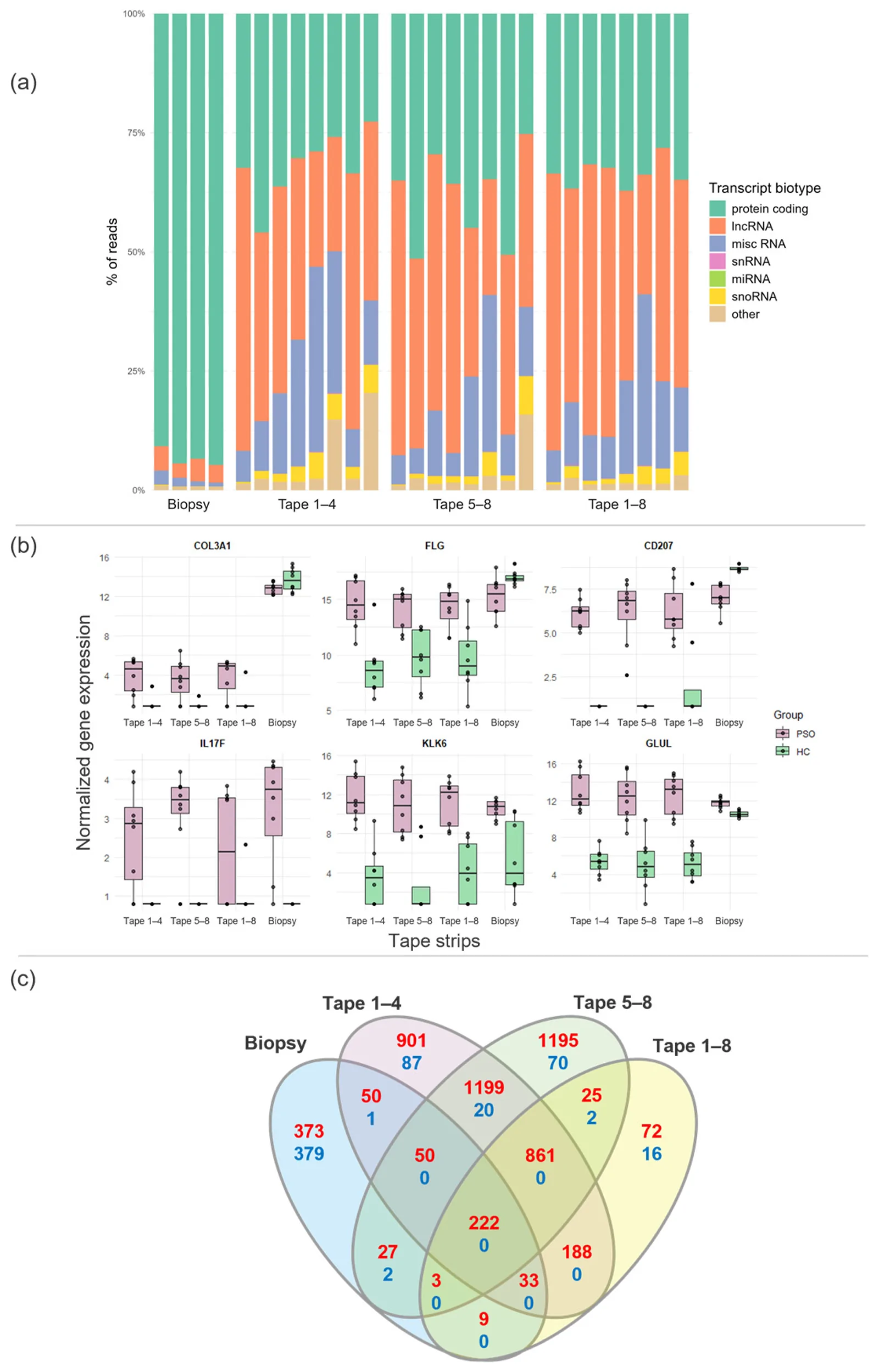
Comparison of tape strips and biopsy specimens from patients with psoriasis. (**a**) Relative biotype distributions of mapped reads from each sample, grouped by sample type. (**b**) Boxplots depicting gene expressions of selected genes in tape strips and biopsies from patients with psoriasis and healthy controls. (**c**) Venn diagram depicting the number of differentially expressed genes (DEGs, FC > 1.5; Padj < 0.05) in different tape strips (1–4, 5–8, and 1–8) and biopsy specimens from patients with psoriasis compared to healthy controls. Upregulated DEGs are red and downregulated DEGs are blue. Abbreviations: HC, healthy controls; lncRNA, long noncoding RNA; miRNA, microRNA; misc RNA, miscellaneous RNA; PSO, psoriasis; snRNA, small nuclear RNA; snoRNA, small nucleolar RNA.

**Table 1:** Baseline characteristics.

	Total	AD	CD	PSO	HC
	n = 32	n = 8	n = 8	n = 8	n = 8
**Age, median (IQR)**	47.0 (24.5)	38.0 (25.0)	57.0 (11.5)	47.5 (21.8)	35.0 (19.3)
**Sex, females, n (%)**	15 (46.9)	4 (50.0)	5 (62.5)	2 (25.0)	4 (50.0)
**Topical treatment ^a^, n (%)**	9 (37.5)	4 (50.0)	3 (37.5)	2 (25.0)	N/A
**Systemic treatment ^b^, n (%)**	13 (54.2)	5 (62.5)	4 (50.0)	4 (50.0)	N/A
**Sample site local severity score for AD ^c^, median (IQR)**	N/A	6.0 (3.5)	N/A	N/A	N/A
**Range 0–12**					
**Sample site local severity score for CD ^d^, median (IQR)**	N/A	N/A	7.5 (2.75)	N/A	N/A
**Range 0–18**					
**Sample site local severity score for PSO ^e^, median (IQR)**	N/A	N/A	N/A	7.0 (1.75)	N/A
**Range 0–12**					
**Sample site ^f^, n (%)**					
Back	7 (21.9)	2 (25.0)	-	2 (25.0)	3 (37.5)
Arm	10 (31.3)	4 (50.0)	1 (12.5)	3 (37.5)	2 (25.0)
Palm	6 (18.8)	-	6 (75.0)	-	-
Buttocks	2 (6.3)	-	-	1 (12.5)	1 (12.5)
Leg	6 (18.8)	2 (25.0)	-	2 (25.0)	2 (25.0)
Foot	1 (3.1)	-	1 (12.5)	-	-

^a^ Topical treatment on the sample site within the last week. ^b^ Systemic treatment at the time of sample collection. 9/13 received Methotrexate, 2/13 Azathioprine, 1/13 Acitretin and 1/13 Mycophenolate mofetil. ^c^ Sample site local severity score for AD patients was calculated based on an evaluation scale from 0 to 3 for erythema, edema/papulation, excoriation, and lichenification, resulting in a score from 0 to 12. ^d^ Sample site local severity score for CD patients was calculated based on an evaluation from 0 to 3 for erythema, infiltration, vesicles, fissures, scaling, and edema, resulting in a score from 0 to 18. ^e^ Sample site local severity score for psoriasis patients was calculated based on an evaluation scale from 0 to 4 for erythema, induration, and scaling, resulting in a score from 0 to 12. ^f^ The sample sites listed are for tape strips. Biopsy specimens were collected from psoriasis patients (arm n = 1, leg n = 2, back n = 1) and healthy controls (leg n = 2, back n = 1, buttocks n = 1). Abbreviations: AD, atopic dermatitis; CD, contact dermatitis; HC, healthy controls; IQR, interquartile range; N/A, not available; PSO, psoriasis.

## Data Availability

The dataset generated in the current study is available from the corresponding author upon reasonable request and pending necessary institutional and ethical approvals.

## Supplementary Materials

The following supporting information can be downloaded at https://www.mdpi.com/article/10.3390/ijms27198544/s1.

## Abbreviations

The following abbreviations are used in this manuscript:

AD	Atopic dermatitis
ACD	Allergic contact dermatitis
CD	Contact dermatitis
ICD	Irritant contact dermatitis
PSO	Psoriasis
HC	Healthy controls
DEG(s)	Differentially expressed gene(s)
FC	Fold change
FDR	False discovery rate
PC	Principal component
PCA	Principal component analysis
GSVA	Gene set variation analysis
GO	Gene ontology
RIN(e)	RNA integrity number (equivalent)
NPX	Normalized protein expression (Olink unit)
WTS	Whole transcriptome sequencing
IQR	Interquartile range
RNA	Ribonucleic acid
lncRNA	Long non-coding RNA
miRNA	MicroRNA
snRNA	Small nuclear RNA
snoRNA	Small nucleolar RNA
