# Supplementary material for: Non-Invasive Transcriptomic Profiling of Inflammatory Skin Diseases Using Four Tape Strips
Source: Int J Mol Sci. 2026 Sep 24;27(19):8544. doi: 10.3390/ijms27198544 (PMC13665526; doi:10.3390/ijms27198544)
Supplement: Supplementary file 1 [file ijms-27-08544-s001.zip › ijms-4564789 - supplementary.pdf]

International Journal of *Molecular Sciences*

MDPI

Supplementary Materials

Sample Collection

Circular tape strips (22 mm, D-Squame, CuDerm, Dallas, Texas, USA) were carefully applied to the skin using a sterile tweezer. Two sets of eight consecutive tape strips were obtained from each participant from two adjacent areas within the same lesion (Figure 1a). At the first site, strips 1–8 were collected sequentially, representing a full-depth stratum corneum sample. At the second site, strips 1–4 and strips 5–8 were collected sequentially but kept separate, representing the superficial and deeper stratum corneum layers, respectively. Each tape strip was applied with standardized pressure for 10 seconds (225 g cm^{-2}) using a pressure applicator (D500, D-Squame, CuDerm). After removal, each tape strip was placed in a sterile 2 mL microtube (SARSTEDT, Nümbrecht, Germany) with the adhesive surface oriented towards the interior of the tube and stored immediately at −80°C until further analysis. Biopsy specimens were placed in 1 mL RNAlater (Thermo Fischer, #AM7020) followed by immediate storage at −80°C.

RNA Isolation

RNA was purified from tape strips using the RNeasy Micro Kit (QIAgen, #74004) and biopsies using RNeasy Fibrous Tissue Mini Kit (Qiagen, #74704) according to the manufacturer's protocols. Briefly, for tapes, 700 µL RLT was added to each tape strip, followed by homogenization using a TissueLyser II (QIAgen). The supernatant was transferred to new Eppendorf tubes and 700 µL ethanol was added. Upon mixture each sample was loaded onto a filter column. The consecutive tapes from each patient (tape 1-8, 1-4 and 5-8) were pooled by adding the lysates one by one to the filter column. For biopsies, samples (<30 mg) were placed in a tube containing 300 µL RLT and homogenized in a TissueLyser II (QIAgen). Tape strips were eluted in 14 µL nuclease-free water while biopsies were eluted in 30 µL. The concentration and purity of the RNA were assessed using a TapeStation 4200 (Agilent) with either the High Sensitivity RNA Screen Tape (Agilent, #5067-5579) or the RNA Screen Tape (Agilent, #5067-5576).

Library preparation and RNA sequencing

Library preparation was performed with the QIAseq® FastSelect™ RNA Library Kit with integrated rRNA removal for complete transcriptome following the manufacturer's instructions (Qiagen, #334235). Briefly, the FastSelect rRNA reagent was directly combined with the RNA and the US RT Buffer (5x), enabling a rapid rRNA removal reaction. cDNA synthesis was performed using the N6-T RT Primer (random hexamer) and the ODT-T RT Primer (oligo-dT primer) for complete transcriptome analysis. Library amplification was performed using the QIAseq UX Index Kits RUDI-96_CA (Qiagen, #331835) and RUDI-96 DA (Qiagen #331845). Quality of individual libraries was assessed using a Tapestation 4200 (Agilent) with High sensitivity DNA screen tape D5000 (Agilent, # 5067-5592) and quantification was assessed using a Qubit 4 Fluorometer (Invitrogen, USA). Samples were normalized and pooled in equimolar concentrations and sequenced (Paired End) on an Illumina NovaSeq 6000 instrument using NovaSeq 6000 S4 Reagent Kit v1.5 (300 cycles) (Illumina, #20028312) and NovaSeq Xp 4-Lane Kit v1.5 (Illumina, #20043131).

Processing of sequencing data, bioinformatic and statistical analysis

RNA sequence reads were processed and mapped to the human reference genome GRCh38 using Qiagen's RNA-seq Analysis Portal 4.0 [31] . Gene name and biotype annotations were retrieved from Ensembl using biomaRt [32] for downstream analyses. All statistical analyses were carried out in R

versions 4.3.0 and 4.4.1. Venn diagrams of the number of detected genes were generated using the vennPlot function from systemPipeR [33] and recreated by hand. A gene was counted as detected if it had an average gene count of at least 10 across the samples. For the remaining analyses, genes were excluded if not present with a read count of at least 10 in four (for biopsies) or eight (for tape strips) samples. Principal component analysis (PCA) was performed on the scaled, centered, and vst-normalized read counts using DESeq2 version 1.42.1 [35] for vst-normalization and the prcomp function from R's stats library to generate the principal components. Variance partitioning was carried out on the vst-normalized counts of protein-coding genes using the variance-Partition library [36].

Differentially expressed genes (DEGs) were identified for each combination of condition (AD, CD, or psoriasis) and sample type (tape strips 1-4, 5-8, 1-8, and for psoriasis, also biopsies) when compared to the corresponding healthy control samples using DESeq2 with an absolute fold change (FC) threshold of 1.5 and 5% false discovery rate (FDR). For the psoriasis biopsies and tape strips, a Venn diagram of the identified DEGs across sample types was created using ggvenn [24] and recreated by hand. Additionally, DEGs between tape strips 1-4 and 5-8 were examined using DESeq2 with default parameters.

Upset plot was generated with the ComplexUpset package [37], [38].

Gene-set variation analysis was performed with the GSVA package on genes with Entrez gene symbols using the gsva approach [39] on gene sets with a minimum size of 10 genes from the Molecular Signatures Database (MSigDB Collection 5: Gene Ontology Biological Process (c5.go.bp.v2025.1)) [40]. We identified differentially expressed pathways at 5% FDR (Benjamini-Hochberg adjustment) in each disease group compared to healthy controls using moderated t-statistics from limma [41]. Furthermore, we compared enrichment scores in each disease group to healthy controls in tape strips 1-4 and 5-8 by investigating the separate contrasts (disease $group_{1\text{-}4}$-$HC_{1\text{-}4}$) and (disease $group_{5\text{-}8}$-$HC_{5\text{-}8}$) along with ((disease $group_{1\text{-}4}$-$HC_{1\text{-}4}$)-(disease $group_{5\text{-}8}$-$HC_{5\text{-}8}$)). A global correction across contrasts was applied to ensure appropriate adjustment for multiple testing.

Some of the study participants were also included in a separate study [18], where tape strips were analyzed using the Olink Target Inflammation panel. Correlations between vst-normalized RNA counts and Olink NPX values were examined per combination of tape strips (1-4, 5-8, and 1-8) for patients who were included in both studies.

Supplementary Figures

Figure S1: Scatterplots of ΔGSVA enrichment scores in pathways from GO Biological Processes when investigating the separate contrasts (disease $group_{1\text{-}4}$-$HC_{1\text{-}4}$), represented on the x-axis, and (disease $group_{5\text{-}8}$-$HC_{5\text{-}8}$), represented on the y-axis, in patients with atopic dermatitis (a), contact dermatitis (b) and psoriasis (c). Dotted diagonal line represents equal effect sizes of ΔGSVA in both sample types. Blue dots are pathways with larger effect size found in the contrast (disease $group_{1\text{-}4}$-$HC_{1\text{-}4}$), red dots are pathways with larger effect size found in the contrast (disease $group_{5\text{-}8}$-$HC_{5\text{-}8}$). Grey dots show pathways with higher activity in healthy controls.

a) Atopic dermatitis vs healthy controls

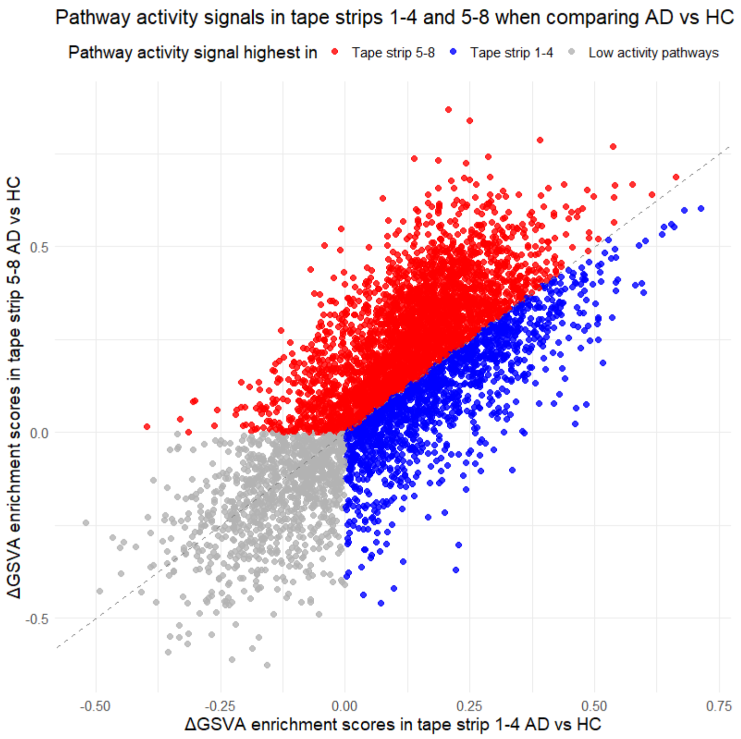

b) Contact dermatitis vs. healthy controls

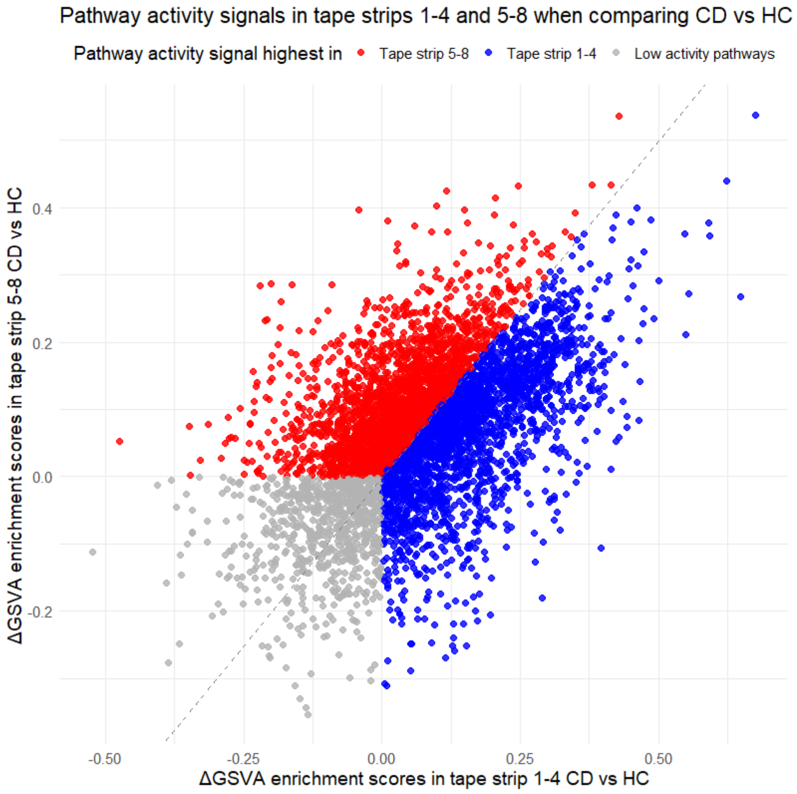

c) Psoriasis vs. healthy controls

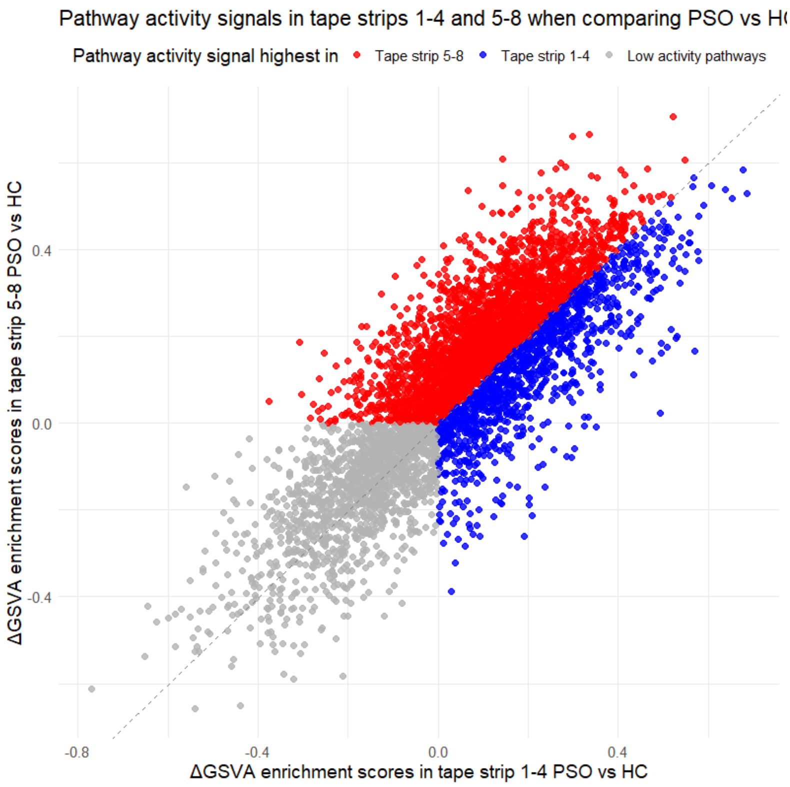

Figure S2: Scatterplot of genes detected (defined as raw count ≥ 10) versus QIAGEN RNA integrity number equivalent (RINe) per WTS sample. Colors and shapes represent the sample type (biopsy, tape) and condition (HC: healthy controls, AD: atopic dermatitis, CD: contact dermatitis, and PSO: psoriasis) associated with a sample. The solid line represents a locally estimated scatterplot smoothing (LOESS) curve, with its 95% confidence interval shaded gray. Samples with missing RINe values were not included.

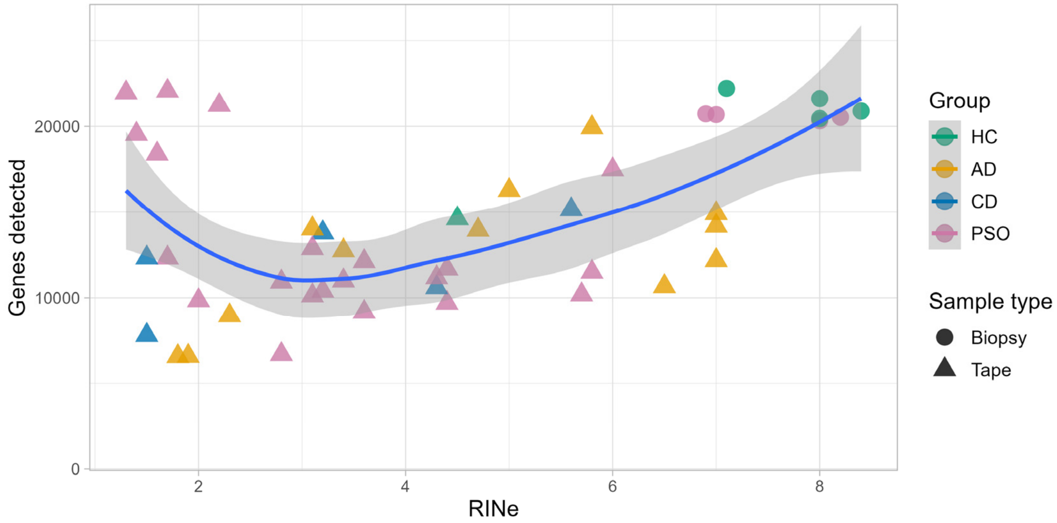

Figure S3: Venn diagram depicting the number of detected genes (mean n ≥ 10) in different tape strips (1-4, 5-8, and 1-8, n=8 in each sample type) and biopsy specimens (n=4) from patients with psoriasis.

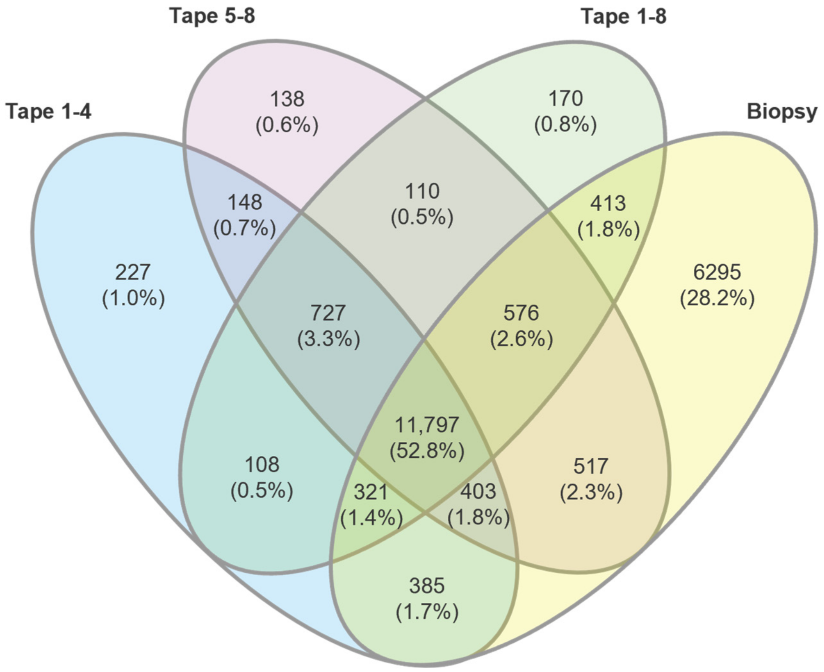

Figure S4: Boxplots of per-sample Pearson correlations in each condition group (AD, CD, PSO, HC) between vst-transformed RNA counts and Olink NPX values from Vinge A.S. et al. [18] of 87 common genes or proteins across different tape strip combinations (1-4, 5-8, and 1-8).

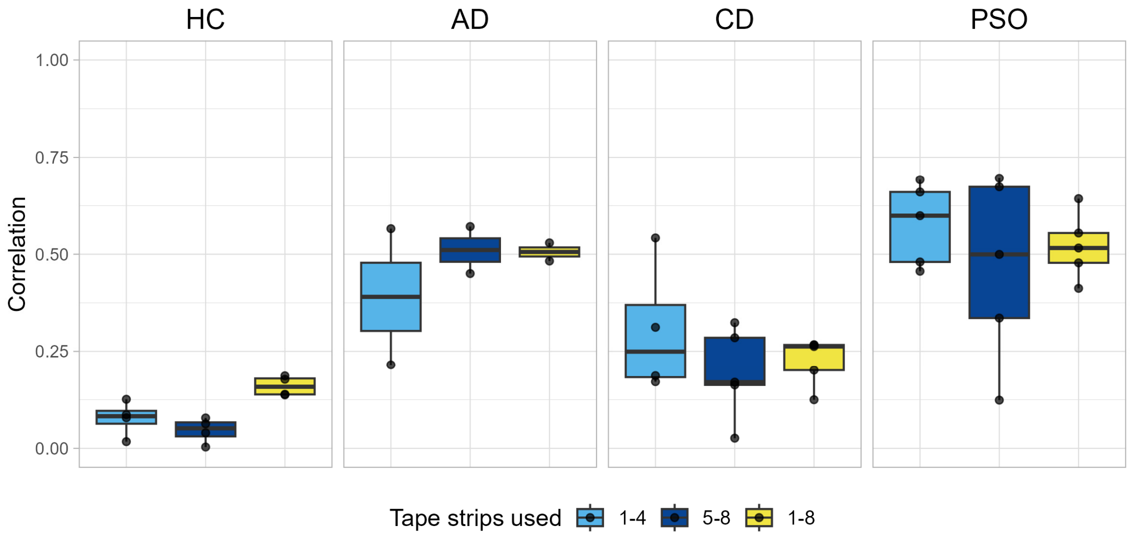

Supplementary Tables

Table S1: Median (IQR) of RNA concentration, reads mapped, and genes detected for tape 1-4, 5-8, and 1-8 from patients with AD, CD, psoriasis, and healthy controls

Table S2: Gene IDs and gene names for Venn diagram of genes detected in tape strips 1-4, 5-8, or 1-8 from patients with AD

Table S3: Gene IDs and gene names for Venn diagram of genes detected in tape strips 1-4, 5-8, or 1-8 from patients with CD

Table S4: Gene IDs and gene names for Venn diagram of genes detected in tape strips 1-4, 5-8, or 1-8 from patients with psoriasis

Table S5: GSVA enrichment scores of Gene Ontology Biological Processes pathways in patients with AD (all tapes) compared to healthy controls

Table S6: GSVA enrichment scores of Gene Ontology Biological Processes pathways in patients with CD (all tapes) compared to healthy controls

Table S7: GSVA enrichment scores of Gene Ontology Biological Processes pathways in patients with psoriasis (all tapes) compared to healthy controls

Table S8: Differences in GSVA enrichment scores (PSO-HC) of Gene Ontology Biological Processes pathways in tape strips 1-4 and 5-8

Table S9: Differences in GSVA enrichment scores (AD-HC) of Gene Ontology Biological Processes pathways in tape strips 1-4 and 5-8

Table S10: Differences in GSVA enrichment scores (CD-HC) of Gene Ontology Biological Processes pathways in tape strips 1-4 and 5-8

Table S11: Relative biotype distributions of mapped reads in tape strips 1-4, 5-8, 1-8, and biopsy specimens from patients with psoriasis

Table S12: Gene IDs and gene names for Venn diagram of genes detected in tape strips 1-4, 5-8, 1-8, and biopsy specimens from patients with psoriasis

Table S13: Gene IDs and gene names for upregulated genes in Venn diagram of DEGs in tape strips 1-4, 5-8, 1-8 and biopsy specimens from patients with psoriasis

Table S14: Gene IDs and gene names for downregulated genes in Venn diagram of DEGs in tape strips 1-4, 5-8, 1-8 and biopsy specimens from patients with psoriasis
